# Supplementary material for: The Nutritional Status of Adult Antiretroviral Therapy Recipients with a Recent HIV Diagnosis; A Cross-Sectional Study in Primary Health Facilities in Gauteng, South Africa
Source: Healthcare (Basel). 2020 Aug 24;8(3):290. doi: 10.3390/healthcare8030290 (PMC7551417; doi:10.3390/healthcare8030290)
Supplement: Supplementary file 1 [file healthcare-08-00290-s001.pdf]

QUESTIONNAIRE

PROJECT TITLE

NUTRITIONAL KNOWLEDGE AND DIETARY PRACTICES OF PEOPLE ON ANTIRETROVIRAL THERAPY IN TSHWANE SUB-DISTRICT 1, GAUTENG PROVINCE

Greetings to participants

Introduction by the researcher

**Participants information**	**Fill in the information**
Date of participation	
Participants' code number	
Contact number	
Name of the facility	
Name of the area	
**SECTION A**<br>**Age and anthropometric measurements**	**Fill in the age, gender and measurements**
1.1. Age (years)	
1.2. Gender	Male ☐<br>Female ☐
1.3. Weight (kg)	1…………<br>2…………<br>3………..
1.4. Height (m)	1……….<br>2……….<br>3………..
1.5. Waist Circumference (cm)	……………
1.6. Hip Circumference (cm)	……………
**SECTION B**<br>**Antiretroviral use**	**Please tick the correct answer, Or fill the answer**
1.7. Are you currently using antiretroviral treatment?	Yes ☐<br>No ☐

1.8. How long have you been on antiretroviral treatment? (in months)	…………….
**SECTION C**<br>**Socio-demographic information**	**Please tick the correct answer, Or fill the answer**
1.9. Marital status	Single ☐<br>Cohabiting ☐<br>Ever married ☐
1.10. Education level	Primary ☐<br>Secondary ☐<br>Completed Grade 12 ☐<br>Tertiary ☐
1.11. Employment status	Yes ☐<br>No ☐
1.12. Receiving social grant	Yes ☐<br>No ☐
1.13. Household monthly income	<R5000 ☐<br>R5001 – R10 000 ☐<br>>R10 001 ☐
1.14. Number of household members	………………..

Thank you,

Researcher’s name
